# 1-Benzoyl-3-(4-chloro­phen­yl)thio­urea dichloro­methane hemisolvate

**DOI:** 10.1107/S1600536812045588

**Published:** 2012-11-10

**Authors:** N. Selvakumaran, M. Mary Sheeba, R. Karvembu, Seik Weng Ng, Edward R. T. Tiekink

**Affiliations:** a Department of Chemistry, National Institute of Technology, Tiruchirappalli 620 015, India; b Department of Chemistry, University of Malaya, 50603 Kuala Lumpur, Malaysia; c Chemistry Department, Faculty of Science, King Abdulaziz University, PO Box 80203 Jeddah, Saudi Arabia

## Abstract

In the title hemisolvate, C_14_H_11_ClN_2_OS·0.5CH_2_Cl_2_, an *anti* disposition is found for the thione and ketone atoms, as well as the N—H H atoms; the dichloro­methane C atom lies on a twofold axis. The central chromophore (including the two adjacent *ipso* C atoms) is planar (r.m.s. deviation = 0.021 Å) owing to the presence of an intra­molecular N—H⋯O hydrogen bond, which closes an *S*(6) loop. Significant twists are evident in the mol­ecule, the dihedral angles between the central moiety and the phenyl and benzene rings being 29.52 (7) and 40.02 (7)°, respectively. In the crystal, eight-membered {⋯HNC= S}_2_ synthons with twofold symmetry form *via* N—H⋯S hydrogen bonds. The dimers are connected into a supra­molecular chain along [111] by C—H⋯O inter­actions. The chains stack along the *c* axis, forming columns which define channels in which the occluded dichloro­methane mol­ecules reside.

## Related literature

For complexation of *N*-benzoyl-*N*′-aryl­thio­urea derivatives to transition metals, see: Selvakumaran *et al.* (2011 ▶). For related structures, see: Khawar Rauf *et al.* (2006 ▶); Selvakumaran *et al.* (2012 ▶).
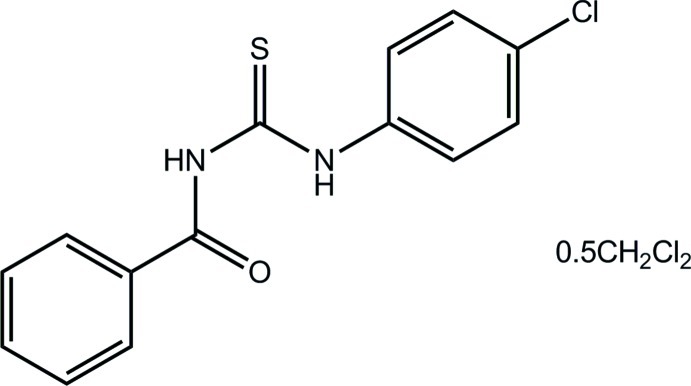


## Experimental

### 

#### Crystal data

C_14_H_11_ClN_2_OS·0.5CH_2_Cl_2_
*M* *_r_* = 333.23
Monoclinic, 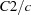

*a* = 20.0800 (4) Å
*b* = 16.0136 (2) Å
*c* = 10.3752 (2) Å
β = 117.690 (3)°
*V* = 2954.10 (9) Å^3^
*Z* = 8
Cu *K*α radiation
μ = 5.26 mm^−1^
*T* = 100 K
0.30 × 0.25 × 0.20 mm

#### Data collection

Agilent SuperNova Dual diffractometer with an Atlas detector
Absorption correction: multi-scan (*CrysAlis PRO*; Agilent, 2012 ▶) *T*_min_ = 0.619, *T*_max_ = 1.000
5766 measured reflections
2937 independent reflections
2802 reflections with *I* > 2σ(*I*)
*R*_int_ = 0.013

#### Refinement

*R*[*F* ^2^ > 2σ(*F* ^2^)] = 0.031
*wR*(*F* ^2^) = 0.090
*S* = 1.05
2937 reflections
194 parameters
H atoms treated by a mixture of independent and constrained refinement
Δρ_max_ = 0.32 e Å^−3^
Δρ_min_ = −0.63 e Å^−3^

### 

Data collection: *CrysAlis PRO* (Agilent, 2012 ▶); cell refinement: *CrysAlis PRO*; data reduction: *CrysAlis PRO*; program(s) used to solve structure: *SHELXS97* (Sheldrick, 2008 ▶); program(s) used to refine structure: *SHELXL97* (Sheldrick, 2008 ▶); molecular graphics: *ORTEP-3 for Windows* (Farrugia, 2012 ▶), *DIAMOND* (Brandenburg, 2006 ▶) and *Qmol* (Gans & Shalloway, 2001 ▶); software used to prepare material for publication: *publCIF* (Westrip, 2010 ▶).

## Supplementary Material

Crystal structure: contains datablock(s) global, I. DOI: 10.1107/S1600536812045588/hg5268sup1.cif

Structure factors: contains datablock(s) I. DOI: 10.1107/S1600536812045588/hg5268Isup2.hkl

Supplementary material file. DOI: 10.1107/S1600536812045588/hg5268Isup3.cml

Additional supplementary materials:  crystallographic information; 3D view; checkCIF report

## Figures and Tables

**Table 1:** Hydrogen-bond geometry (Å, °)

*D*—H⋯*A*	*D*—H	H⋯*A*	*D*⋯*A*	*D*—H⋯*A*
N2—H2*n*⋯O1	0.90 (2)	1.85 (2)	2.6034 (17)	140.6 (18)
N1—H1*n*⋯S1^i^	0.87 (2)	2.59 (2)	3.4368 (15)	167 (2)
C12—H12⋯O1^ii^	0.95	2.47	3.3743 (19)	160

Symmetry codes: (i) 
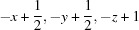
; (ii) 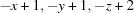.

**Fig. 1.**
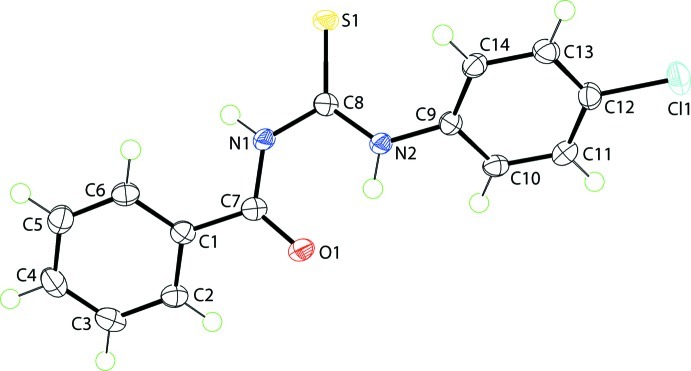
Molecular structure of (I) showing atom-labelling scheme and displacement ellipsoids at the 70% probability level. The dichloromethane molecule of solvation has been omitted.

**Fig. 2.**
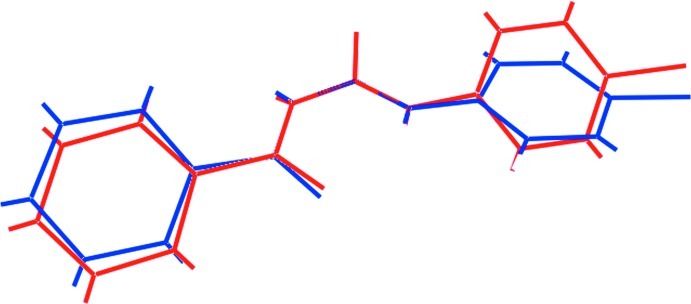
Overlay diagram of N-benzoyl-N'-arylthiourea derivative (I) with the unsolvated form (blue). The NSN atoms have been superimposed.

**Fig. 3.**
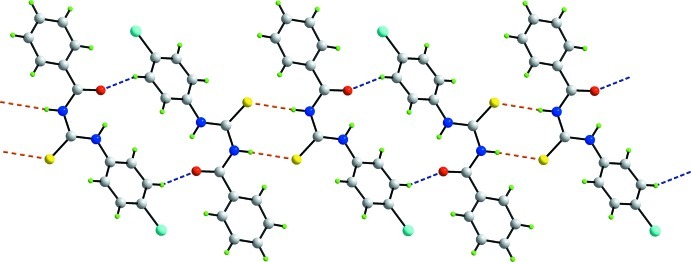
A view of the supramolecular chain along [111] in (I). The N—H···S and C—H···O interactions are shown as orange and blue dashed lines, respectively.

**Fig. 4.**
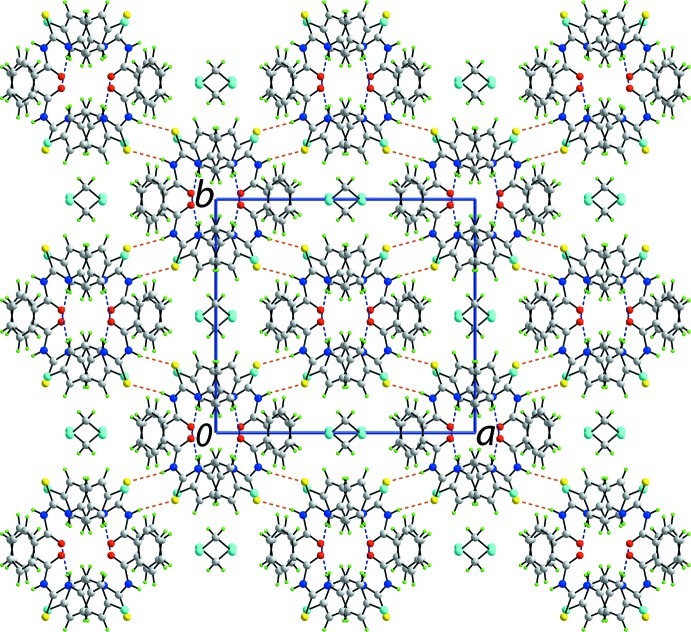
A view of the unit-cell contents in projection down the c axis in (I). The N—H···S and C—H···O interactions are shown as orange and blue dashed lines, respectively.

## supplementary crystallographic information

### Comment

As part of continuing studies of *N*-benzoyl-*N*'-arylthiourea derivatives (Selvakumaran *et al.*, 2012) and their coordination to transition metals such as Pd^II^ (Selvakumaran *et al.*, 2011), the title compound (I) was investigated as its dichloromethane hemi-solvate. The structure of the unsolvated form is available for comparison (Khawar Rauf *et al.*, 2006).

In (I), Fig. 1, *anti* dispositions are found for the thione and ketone atoms, and for the N—H H-atoms. The central chromophore (including the two adjacent *ipso* C atoms) is planar with a r.m.s. deviation of 0.021 Å [maximum deviations of 0.033 (1) Å for N1, and -0.023 (1) Å for S1] owing to the presence of an intramolecular N—H···O hydrogen bond which closes an *S*(6) loop, Table 1. The observed disposition of atoms is the same as for the unsolvated form (Khawar Rauf *et al.*, 2006).

A significant twist is evident in (I) as seen in the dihedral angles of 29.52 (7) and 40.02 (7)° formed between the central moiety and the C1-phenyl and C9-benzene rings, respectively. The dihedral angle between the six-membered rings is 11.11 (9)°. This pattern of dihedral angles contrasts the situation for the unsolvated form where the comparable dihedral angles are 41.42 (8), 2.77 (8) and 43.93 (10)°, respectively, indicating a significantly greater twist in the molecule as highlighted in the overlay diagram, Fig. 2.

Eight-membered {···HNC═S}_2_ synthons (2-fold symmetry) feature in the crystal packing which arise *via* N—H···S hydrogen bonds and these are connected into a supramolecular chain along [111] by C—H···O interactions which stabilize centrosymmetric 18-membered {···OCNCNC_3_H}_2_ synthons., Fig. 3 and Table 1. Chains stack along the *c* axis to form columns which define channels in which the occluded dichloromethane molecules (with 2-fold axis symmetry) reside. There are no specific interactions between the chains nor with the solvent molecules, Fig. 4.

### Experimental

A solution of benzoyl chloride (0.005 mol, 0.7029 g) in acetone (30 ml) was added drop wise to a suspension of potassium thiocyanate (0.005 mol, 0.4859 g) in anhydrous acetone (30 ml). The reaction mixture was heated under reflux for 45 minutes and then cooled to room temperature. A solution of '4-chloroaniline (0.005 mol, 0.6379 g) in acetone (30 ml) was added and the resulting mixture was stirred for 2 h. Hydrochloric acid (0.1 N, 300 ml) was added and resulting solid was filtered, washed with water and dried *in vacuo*. The resulting solid product (78% yield) was recrystallized as colourless blocks from its ethanol/dichloromethane (1:2 ratio) solution. Characterization (dried sample): *M*.pt: 411 K, Anal. Calcd. For C_14_H_11_ClN_2_O_2_S (%): C, 57.8; H, 3.81; N, 9.63; Found: C, 57.5; H, 4.0; N, 9.8. ^1^H NMR: δ (400 MHz, CDCl_3_, p.p.m.): 7.35–7.88 (*m*, 9H); 9.14 (*s*, 1H, thiourea NH); 12.58 (*s*, 1H, amide NH). ^13^C NMR: δ (400 MHz, CDCl_3_, p.p.m.): 125.3; 127.5; 129.0; 129.2; 131.4; 132.1; 133.8; 136.1; 167.0; 178.4. IR (KBr, cm^-1^): 3230 ν(amide N–H), 3028 ν(thiourea N–H), 1688 ν(C=O), 1262 ν(C=S).

### Refinement

Carbon-bound H-atoms were placed in calculated positions [C—H = 0.95 to 0.99 Å, *U*_iso_(H)= 1.2 to 1.5*U*_eq_(C)] and were included in the refinement in the riding model approximation. The N-bound H-atoms were refined freely. Despite the elongated anisotropic displacement parameters for the dichloromethane-C atom, multiple positions could not be resolved.

### Crystal data

**Table:**

C_14_H_11_ClN_2_OS·0.5CH_2_Cl_2_	*F*(000) = 1368
*M**_r_* = 333.23	*D*_x_ = 1.498 Mg m^−^^3^
Monoclinic, *C*2/*c*	Cu *K*α radiation, λ = 1.54184 Å
Hall symbol: -C 2yc	Cell parameters from 3963 reflections
*a* = 20.0800 (4) Å	θ = 3.7–74.2°
*b* = 16.0136 (2) Å	µ = 5.26 mm^−^^1^
*c* = 10.3752 (2) Å	*T* = 100 K
β = 117.690 (3)°	Block, colourless
*V* = 2954.10 (9) Å^3^	0.30 × 0.25 × 0.20 mm
*Z* = 8	

### Data collection

**Table:**

Agilent SuperNova Dual diffractometer with an Atlas detector	2937 independent reflections
Radiation source: SuperNova (Cu) X-ray Source	2802 reflections with *I* > 2σ(*I*)
Mirror monochromator	*R*_int_ = 0.013
Detector resolution: 10.4041 pixels mm^-1^	θ_max_ = 74.4°, θ_min_ = 3.7°
ω scan	*h* = −19→24
Absorption correction: multi-scan (*CrysAlis PRO*; Agilent, 2012)	*k* = −19→18
*T*_min_ = 0.619, *T*_max_ = 1.000	*l* = −12→8
5766 measured reflections	

### Refinement

**Table:**

Refinement on *F*^2^	Primary atom site location: structure-invariant direct methods
Least-squares matrix: full	Secondary atom site location: difference Fourier map
*R*[*F*^2^ > 2σ(*F*^2^)] = 0.031	Hydrogen site location: inferred from neighbouring sites
*wR*(*F*^2^) = 0.090	H atoms treated by a mixture of independent and constrained refinement
*S* = 1.05	*w* = 1/[σ^2^(*F*_o_^2^) + (0.0515*P*)^2^ + 3.8195*P*] where *P* = (*F*_o_^2^ + 2*F*_c_^2^)/3
2937 reflections	(Δ/σ)_max_ = 0.001
194 parameters	Δρ_max_ = 0.32 e Å^−^^3^
0 restraints	Δρ_min_ = −0.63 e Å^−^^3^

### Special details

**Table:**

Geometry. All e.s.d.'s (except the e.s.d. in the dihedral angle between two l.s. planes) are estimated using the full covariance matrix. The cell e.s.d.'s are taken into account individually in the estimation of e.s.d.'s in distances, angles and torsion angles; correlations between e.s.d.'s in cell parameters are only used when they are defined by crystal symmetry. An approximate (isotropic) treatment of cell e.s.d.'s is used for estimating e.s.d.'s involving l.s. planes.
Refinement. Refinement of *F*^2^ against ALL reflections. The weighted *R*-factor *wR* and goodness of fit *S* are based on *F*^2^, conventional *R*-factors *R* are based on *F*, with *F* set to zero for negative *F*^2^. The threshold expression of *F*^2^ > σ(*F*^2^) is used only for calculating *R*-factors(gt) *etc*. and is not relevant to the choice of reflections for refinement. *R*-factors based on *F*^2^ are statistically about twice as large as those based on *F*, and *R*- factors based on ALL data will be even larger.

### Fractional atomic coordinates and isotropic or equivalent isotropic displacement parameters (Å^2^)

**Table:**

	*x*	*y*	*z*	*U*_iso_*/*U*_eq_	Occ. (<1)
Cl1	0.64817 (3)	0.24103 (3)	1.38168 (4)	0.02772 (13)	
Cl2	0.06334 (3)	0.49157 (3)	0.21781 (6)	0.04144 (16)	
S1	0.34276 (2)	0.21105 (2)	0.68579 (4)	0.01812 (12)	
O1	0.40404 (6)	0.47474 (7)	0.61554 (12)	0.0182 (2)	
N2	0.43155 (7)	0.34562 (8)	0.78527 (14)	0.0141 (3)	
N1	0.33686 (7)	0.35253 (8)	0.54885 (14)	0.0139 (3)	
C1	0.30153 (8)	0.46811 (10)	0.37713 (17)	0.0142 (3)	
C2	0.26545 (9)	0.41884 (10)	0.25243 (17)	0.0161 (3)	
H2	0.2727	0.3601	0.2585	0.019*	
C3	0.21909 (9)	0.45587 (11)	0.11989 (18)	0.0196 (3)	
H3	0.1946	0.4224	0.0350	0.024*	
C4	0.20837 (9)	0.54186 (11)	0.11089 (18)	0.0210 (3)	
H4	0.1760	0.5669	0.0201	0.025*	
C5	0.24478 (9)	0.59118 (11)	0.23394 (19)	0.0199 (3)	
H5	0.2374	0.6499	0.2271	0.024*	
C6	0.29191 (9)	0.55510 (10)	0.36699 (18)	0.0166 (3)	
H6	0.3176	0.5891	0.4509	0.020*	
C7	0.35206 (8)	0.43302 (10)	0.52309 (17)	0.0143 (3)	
C8	0.37385 (8)	0.30647 (10)	0.67746 (17)	0.0135 (3)	
C9	0.48080 (8)	0.31534 (10)	0.92652 (17)	0.0136 (3)	
C10	0.50405 (8)	0.37306 (10)	1.03984 (17)	0.0151 (3)	
H10	0.4840	0.4280	1.0210	0.018*	
C12	0.55623 (9)	0.35074 (10)	1.17967 (17)	0.0166 (3)	
H12	0.5727	0.3901	1.2568	0.020*	
C13	0.58394 (9)	0.26988 (11)	1.20494 (18)	0.0172 (3)	
C14	0.56186 (9)	0.21168 (10)	1.09322 (18)	0.0179 (3)	
H14	0.5820	0.1567	1.1126	0.021*	
C15	0.51002 (9)	0.23468 (10)	0.95287 (17)	0.0156 (3)	
H15	0.4946	0.1956	0.8754	0.019*	
C16	0.0000	0.5521 (2)	0.2500	0.090 (2)	
H16A	−0.0286	0.5885	0.1647	0.108*	0.50
H16B	0.0286	0.5885	0.3353	0.108*	0.50
H1n	0.2952 (13)	0.3301 (14)	0.485 (2)	0.026 (6)*	
H2n	0.4380 (12)	0.3990 (15)	0.767 (2)	0.029 (6)*	

### Atomic displacement parameters (Å^2^)

**Table:**

	*U*^11^	*U*^22^	*U*^33^	*U*^12^	*U*^13^	*U*^23^
Cl1	0.0299 (2)	0.0251 (2)	0.0164 (2)	0.00383 (17)	0.00079 (18)	0.00311 (15)
Cl2	0.0391 (3)	0.0343 (3)	0.0530 (3)	0.0115 (2)	0.0232 (3)	0.0051 (2)
S1	0.0128 (2)	0.0128 (2)	0.0220 (2)	−0.00272 (13)	0.00228 (16)	0.00305 (14)
O1	0.0162 (5)	0.0142 (5)	0.0196 (6)	−0.0034 (4)	0.0045 (5)	−0.0006 (4)
N2	0.0115 (6)	0.0118 (6)	0.0156 (7)	−0.0008 (5)	0.0035 (5)	0.0006 (5)
N1	0.0102 (6)	0.0128 (6)	0.0148 (6)	−0.0013 (5)	0.0027 (5)	−0.0001 (5)
C1	0.0112 (7)	0.0156 (7)	0.0169 (7)	−0.0007 (6)	0.0074 (6)	0.0012 (6)
C2	0.0147 (7)	0.0155 (7)	0.0197 (8)	−0.0019 (6)	0.0096 (6)	−0.0003 (6)
C3	0.0186 (8)	0.0237 (8)	0.0168 (8)	−0.0034 (7)	0.0084 (6)	−0.0018 (7)
C4	0.0182 (8)	0.0254 (9)	0.0186 (8)	0.0022 (7)	0.0079 (7)	0.0078 (7)
C5	0.0189 (8)	0.0163 (8)	0.0264 (8)	0.0017 (6)	0.0123 (7)	0.0057 (7)
C6	0.0157 (7)	0.0150 (7)	0.0199 (8)	−0.0012 (6)	0.0090 (6)	0.0001 (6)
C7	0.0122 (7)	0.0133 (7)	0.0181 (7)	0.0009 (6)	0.0077 (6)	0.0000 (6)
C8	0.0102 (7)	0.0132 (7)	0.0172 (8)	0.0015 (6)	0.0065 (6)	0.0010 (6)
C9	0.0091 (7)	0.0153 (7)	0.0158 (7)	−0.0006 (6)	0.0051 (6)	0.0009 (6)
C10	0.0134 (7)	0.0127 (7)	0.0198 (8)	−0.0006 (6)	0.0082 (6)	−0.0003 (6)
C12	0.0153 (7)	0.0172 (8)	0.0174 (8)	−0.0032 (6)	0.0077 (6)	−0.0035 (6)
C13	0.0140 (7)	0.0198 (8)	0.0152 (7)	−0.0003 (6)	0.0047 (6)	0.0023 (6)
C14	0.0154 (8)	0.0139 (8)	0.0217 (8)	0.0015 (6)	0.0065 (7)	0.0014 (6)
C15	0.0130 (7)	0.0143 (7)	0.0175 (8)	−0.0004 (6)	0.0054 (6)	−0.0021 (6)
C16	0.077 (3)	0.0225 (16)	0.218 (7)	0.000	0.110 (4)	0.000

### Geometric parameters (Å, º)

**Table:**

Cl1—C13	1.7439 (16)	C4—H4	0.9500
Cl2—C16	1.750 (2)	C5—C6	1.386 (2)
S1—C8	1.6679 (16)	C5—H5	0.9500
O1—C7	1.2350 (19)	C6—H6	0.9500
N2—C8	1.335 (2)	C9—C15	1.392 (2)
N2—C9	1.419 (2)	C9—C10	1.395 (2)
N2—H2n	0.90 (2)	C10—C12	1.385 (2)
N1—C7	1.379 (2)	C10—H10	0.9500
N1—C8	1.398 (2)	C12—C13	1.385 (2)
N1—H1n	0.87 (2)	C12—H12	0.9500
C1—C2	1.396 (2)	C13—C14	1.390 (2)
C1—C6	1.403 (2)	C14—C15	1.390 (2)
C1—C7	1.487 (2)	C14—H14	0.9500
C2—C3	1.385 (2)	C15—H15	0.9500
C2—H2	0.9500	C16—Cl2^i^	1.750 (2)
C3—C4	1.390 (2)	C16—H16A	0.9900
C3—H3	0.9500	C16—H16B	0.9900
C4—C5	1.386 (3)		
			
C8—N2—C9	128.44 (14)	N2—C8—N1	114.91 (13)
C8—N2—H2n	115.0 (14)	N2—C8—S1	125.93 (12)
C9—N2—H2n	116.4 (14)	N1—C8—S1	119.15 (11)
C7—N1—C8	127.64 (13)	C15—C9—C10	120.27 (14)
C7—N1—H1n	117.6 (15)	C15—C9—N2	123.21 (14)
C8—N1—H1n	113.8 (15)	C10—C9—N2	116.34 (14)
C2—C1—C6	119.81 (15)	C12—C10—C9	120.40 (15)
C2—C1—C7	123.01 (14)	C12—C10—H10	119.8
C6—C1—C7	117.16 (14)	C9—C10—H10	119.8
C3—C2—C1	119.86 (15)	C10—C12—C13	118.77 (15)
C3—C2—H2	120.1	C10—C12—H12	120.6
C1—C2—H2	120.1	C13—C12—H12	120.6
C2—C3—C4	120.15 (15)	C12—C13—C14	121.64 (15)
C2—C3—H3	119.9	C12—C13—Cl1	118.83 (13)
C4—C3—H3	119.9	C14—C13—Cl1	119.53 (13)
C5—C4—C3	120.25 (15)	C13—C14—C15	119.32 (15)
C5—C4—H4	119.9	C13—C14—H14	120.3
C3—C4—H4	119.9	C15—C14—H14	120.3
C4—C5—C6	120.24 (15)	C14—C15—C9	119.59 (15)
C4—C5—H5	119.9	C14—C15—H15	120.2
C6—C5—H5	119.9	C9—C15—H15	120.2
C5—C6—C1	119.67 (15)	Cl2—C16—Cl2^i^	112.8 (2)
C5—C6—H6	120.2	Cl2—C16—H16A	109.0
C1—C6—H6	120.2	Cl2^i^—C16—H16A	109.0
O1—C7—N1	122.58 (14)	Cl2—C16—H16B	109.0
O1—C7—C1	121.13 (14)	Cl2^i^—C16—H16B	109.0
N1—C7—C1	116.29 (13)	H16A—C16—H16B	107.8
			
C6—C1—C2—C3	−1.4 (2)	C9—N2—C8—S1	2.8 (2)
C7—C1—C2—C3	179.99 (14)	C7—N1—C8—N2	−1.7 (2)
C1—C2—C3—C4	−0.1 (2)	C7—N1—C8—S1	177.10 (12)
C2—C3—C4—C5	0.9 (2)	C8—N2—C9—C15	41.7 (2)
C3—C4—C5—C6	−0.3 (2)	C8—N2—C9—C10	−143.19 (16)
C4—C5—C6—C1	−1.2 (2)	C15—C9—C10—C12	−0.2 (2)
C2—C1—C6—C5	2.0 (2)	N2—C9—C10—C12	−175.41 (13)
C7—C1—C6—C5	−179.27 (14)	C9—C10—C12—C13	−0.9 (2)
C8—N1—C7—O1	1.6 (2)	C10—C12—C13—C14	1.4 (2)
C8—N1—C7—C1	−177.64 (14)	C10—C12—C13—Cl1	−178.67 (12)
C2—C1—C7—O1	151.52 (15)	C12—C13—C14—C15	−0.8 (2)
C6—C1—C7—O1	−27.2 (2)	Cl1—C13—C14—C15	179.28 (12)
C2—C1—C7—N1	−29.2 (2)	C13—C14—C15—C9	−0.3 (2)
C6—C1—C7—N1	152.07 (14)	C10—C9—C15—C14	0.8 (2)
C9—N2—C8—N1	−178.51 (14)	N2—C9—C15—C14	175.69 (14)

Symmetry code: (i) −*x*, *y*, −*z*+1/2.

### Hydrogen-bond geometry (Å, º)

**Table:**

*D*—H···*A*	*D*—H	H···*A*	*D*···*A*	*D*—H···*A*
N2—H2*n*···O1	0.90 (2)	1.85 (2)	2.6034 (17)	140.6 (18)
N1—H1*n*···S1^ii^	0.87 (2)	2.59 (2)	3.4368 (15)	167 (2)
C12—H12···O1^iii^	0.95	2.47	3.3743 (19)	160

Symmetry codes: (ii) −*x*+1/2, −*y*+1/2, −*z*+1; (iii) −*x*+1, −*y*+1, −*z*+2.
